# Novel strategy for depolymerization of avermectin fermentation residue to value-added amino acid product

**DOI:** 10.3389/fchem.2024.1375223

**Published:** 2024-03-01

**Authors:** Chi Ma, Zhengxin Mao, Qingfen Liu

**Affiliations:** ^1^ CAS Key Laboratory of Green Process and Engineering, Institute of Process Engineering, Chinese Academy of Sciences, Beijing, China; ^2^ School of Chemical Engineering, University of Chinese Academy of Sciences, Beijing, China

**Keywords:** avermectin fermentation residue, depolymerization, acid hydrolysis, protein, amino acid

## Abstract

Avermectin fermentation residue (AFR) is rich in proteins, which can be depolymerized to value-added amino acids for in-plant reuse. The hydrochloric acid (HCl) hydrolysis is performed and investigated under different conditions, including HCl concentration, solid-liquid ratio, temperature, and time. The hydrolysis degree (HD) of 67.7% can be achieved. The empirical correlation of HD is established with a good practicability to control the HD and predict the experimental conditions. Solid-liquid reaction is confirmed to be dominant during the hydrolysis process. There are 17 kinds of amino acids in the hydrolysate, benefiting the reuse. Avermectin is not detected in the hydrolysate and AFR, and the mass of AFR is reduced by 53.8 wt%. This work provides a novel strategy for the environmentally friendly treatment and meanwhile the resource recovery of AFR.

## 1 Introduction

Avermectin is a 16-membered macrolide antibiotics, which has been recognized as the most produced and used pesticide in the world with remarkable insecticidal, acaricidal and nematicidal activity (Hao et al., 2022; Wang et al., 2023; Xu et al., 2023). Avermectin fermentation residue (AFR) is a byproduct of the fermentation process for producing avermectin, and the annual production of this residue is about 40,000 tons (Cai et al., 2019). AFR mainly consists of mycelial cells, unutilized culture media, microbial metabolites, and residual avermectin (Geng et al., 2023). The residual avermectin presents the potential risks of the spread of antibiotic resistance in organisms (Ding et al., 2019), which seriously threatens the ecosystem and human health (Barra Caracciolo et al., 2011; Huang et al., 2017; Wang et al., 2022). In this context, AFR has been classified as “Hazardous Waste” in China (Hu et al., 2020; Song et al., 2020).

The traditional methods for the treatment of fermentation residues are incineration, safe landfill, and composting (Boukhrissa et al., 2017), but these methods always lead to resource waste and secondary pollution (Han et al., 2014; Christou et al., 2018; Cai et al., 2021). It is imperative to develop an alternative method for the environmentally friendly treatment and meanwhile the resource recovery of AFR. As a biosolid, AFR is rich in organic matter, especially proteins, which are as high as 30–50 wt% (Zhang et al., 2021). Due to the insoluble property, most proteins are difficult to reuse, and the depolymerization process such as acid (Adebiyi et al., 2005; Lopez et al., 2019), alkali (Dabrowska et al., 2022; Solanki et al., 2023), and enzyme hydrolysis (Leni et al., 2020; Pacheco et al., 2023) can be used to release small molecules as nutrient sources (Rezania et al., 2020; Nnaemeka et al., 2021; Chen et al., 2023). Zhang and Zhao. (2017) proposed the hydrolysis of proteins in penicillin fermentation residues to amino acids with sodium hydroxide, achieving an amino acid nitrogen content of 1.8 g/L. Zhang and Liu. (2021) investigated the enzyme hydrolysis of proteins in penicillin fermentation residues to amino acids, and the hydrolysis degree (HD) of proteins was 42.7%.

Some researchers have investigated the depolymerization of proteins in animals and plants with acid medium. Hu et al. (2015) proposed the hydrochloric acid (HCl) hydrolysis of wheat bran proteins to amino acids, showing an HD of 59.2%. Dai et al. (2019) reported the HCl hydrolysis of microalgae proteins for the production of polypeptides, and the maximum HD was 57.0%. Ma et al. (2010) compared the hydrolysis performance of corbicula fluminea proteins between different types of acids. The results showed that HCl was more efficient than citric and lactic acids with a higher HD of 59.3%. Acid medium has the ability to break the peptidoglycan cell walls of AFR and the advantages of low operating cost and high digestibility (Baadhe et al., 2014; Saville et al., 2016; Lin et al., 2022). It is hopeful to apply the acid medium to depolymerize AFR to small molecules for in-plant reuse, avoiding the environmental pollution during the ex-plant treatment. Up to now, there are few reports on the acid hydrolysis of AFR. The hydrolysis performance and mechanism may be different because of the complex composition, which are still unclear.

In this work, a novel strategy for AFR depolymerization with acid medium was proposed, and the HD of proteins in AFR was investigated under different conditions. The correlation of HD was established based on the results of hydrolysis experiments. The dominant reaction was explored through the comparison of the performance between different hydrolysis processes. Moreover, the components in the hydrolysate and avermectin in AFR were analyzed to discuss the feasibility of in-plant reuse.

## 2 Experimental section

**FIGURE 1:**
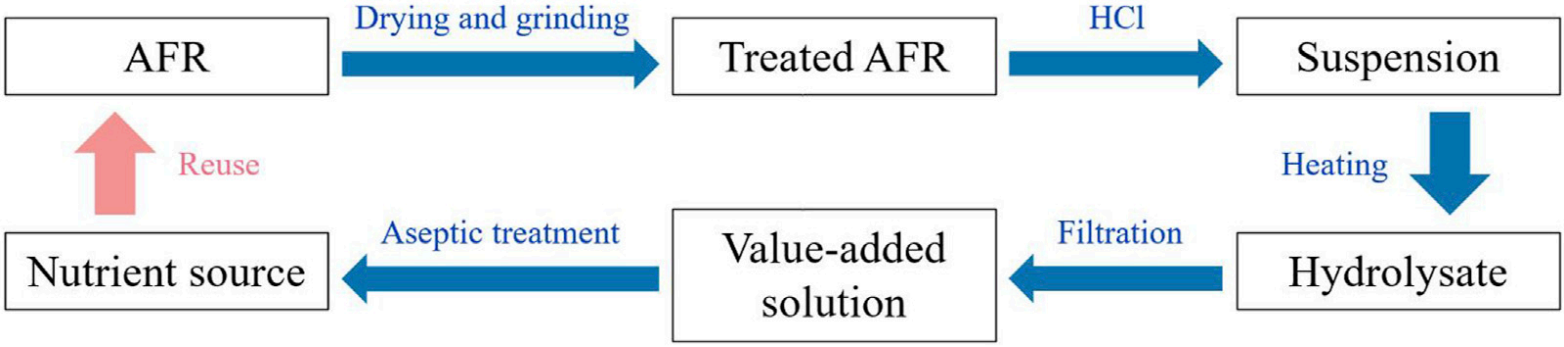
Strategy for AFR depolymerization with acid medium.

In this work, a strategy for AFR depolymerization with acid medium was proposed, as shown in Figure 1. The feasibility of the depolymerization process was evaluated.

### 2.1 Materials and reagents

AFR and avermectin powders were derived from Qilu Pharmaceutical (Inner Mongolia) Co., Ltd., China. HCl, phenol, and sulfuric acid (H_2_SO_4_) were purchased from Sinopharm Chemical Reagent Co., Ltd., China. Methanol was purchased from Thermo Fisher Scientific Inc., the United States. All the reagents were analytically pure and used as received.

### 2.2 Depolymerization of AFR

According to Figure 1, AFR was dried in a drying oven (DHG-9070A, Shanghai Yiheng Scientific Instrument Co., Ltd., China) at 80 °C for 24 h, and then ground to less than 270 μm. HCl was added into deionized water to prepare a solution. A certain mass of treated AFR and a certain volume of HCl solution were introduced into a round bottom flask. The flask was immersed in a water bath (DF-101S, Gongyi Yuhua Instrument Co., Ltd., China) to control the hydrolysis temperature. After the hydrolysis for a period of time, the mixture was naturally cooled to room temperature (25 °C) and allowed to stand for 30 min. The HCl concentration (*C*_HCl_) of 1, 2, 4, 6, and 8 mol/L, solid-liquid ratio (mass:volume, *R*_SL_) of 1:5, 10, 20, 40, and 80 g/mL, hydrolysis temperature (*T*) of 40, 60, 80, 90, 100, 110, and 120 °C, and hydrolysis time (*t*) of 10, 20, 30, 40, 60, 120, 240, and 360 min were chosen for the experiments. Subsequently, the mixture was filtered to achieve solid-liquid separation. The mass of solid and the composition of liquid were measured and recorded, and each experimental condition was repeated for three times. The value-added solution can be aseptically treated for further reuse.

For the investigation of the dominant reaction of hydrolysis process, three experiments were performed under the same HCl concentration, solid-liquid ratio, and hydrolysis temperature. In the first experiment, the hydrolysis time was 120 min, marked as mode #1. The hydrolysis time of the second experiment was set as 30 min. Then the mixture was filtered, and the liquid was continuously hydrolyzed for another 90 min, marked as mode #2. The hydrolysis time of the third experiment and the time for continue hydrolysis of the liquid were set as 60 and 60 min, respectively, marked as mode #3.

### 2.3 Analytical methods

The protein content in AFR and hydrolysate was determined by a Kjeldahl nitrogen analyzer (K06A, Shanghai Chengsheng Automation Analysis Instrument Co., Ltd., China). The fat content in AFR and hydrolysate was determined by a Soxhlet extractor (BSXT-06, Shanghai Hefan Instrument Co., Ltd., China). The ash content in AFR was characterized by a ceramic fiber muffle furnace (TM-0610P, Beijing Yinganmeicheng Scientific Instrument Co., Ltd., China). The amino acid content in the hydrolysate was analyzed by an automatic amino acid analyzer (L-8900, Hitachi Ltd., Japan).

The avermectin content in the hydrolysate was analyzed by a high performance liquid chromatography (HPLC, 1,260, Agilent Technologies Inc., the United States) with a column (ZORBAX SB-C18, 4.6 mm × 250 mm, 5 μm). The detection wavelength of 245 nm, mobile phase (methanol and water) volume ratio of 85:15, flow rate of 1.0 mL/min, injection volume of 20 μL, column temperature of 25°C ± 0.8°C, and retention time of 25 min were used for the chromatographic conditions. The content of avermectin powders with known concentration was used as a reference. For the analysis of avermectin content in AFR, AFR was extracted with methanol at room temperature for 60 min, and the solid-liquid ratio (mass:volume) was set as 1:20 g/mL. The avermectin content in the extract was analyzed by HPLC. The products of avermectin hydrolysis were identified by a high resolution liquid chromatography-mass spectrometry system (LC-MS, TripleTOF 5,600, AB Sciex Pte. Ltd., the United States).

The soluble saccharide content in the hydrolysate was measured by an ultraviolet-visible spectrophotometer (UH5300, Hitachi Ltd., Japan) at a wavelength of 490 nm. The absorbance (Abs) of samples with known concentration was used as a reference. For the measurement of soluble saccharide content in AFR, AFR was added in deionized water, and then 1 mL phenol with concentration of 5 wt% and 5 mL H_2_SO_4_ were added in the liquid, respectively. The mixture was shaken and allowed to stand for 15 min at room temperature and 30°C, respectively. The Abs of the liquid was measured by ultraviolet-visible spectrophotometer.

## 3 Results and discussion

### 3.1 Hydrolysis performance of proteins in AFR

#### 3.1.1 Hydrolysis degree

The protein, fat, ash, soluble saccharide, and avermectin contents in AFR are listed in Table 1. It can be seen that the protein content was high as 300.4 mg/g (30.0 wt%). This indicated that AFR was suitable and valuable for reuse to produce small molecules as nutrient sources.

**TABLE 1:** The protein, fat, ash, soluble saccharide, and avermectin contents in AFR.

Composition	Protein	Fat	Ash	Soluble saccharide	Avermectin
Content (mg/g)	300.4	87.0	63.1	45.1	0.4

**FIGURE 2:**
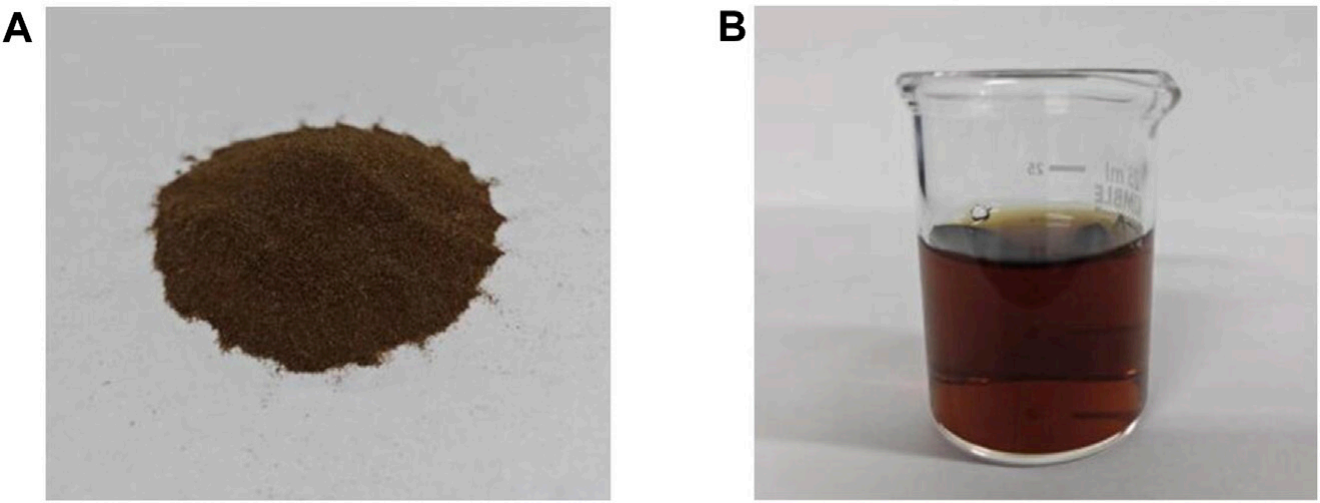
Photographs of AFR **(A)** before and **(B)** after HCl hydrolysis.

**FIGURE 3:**
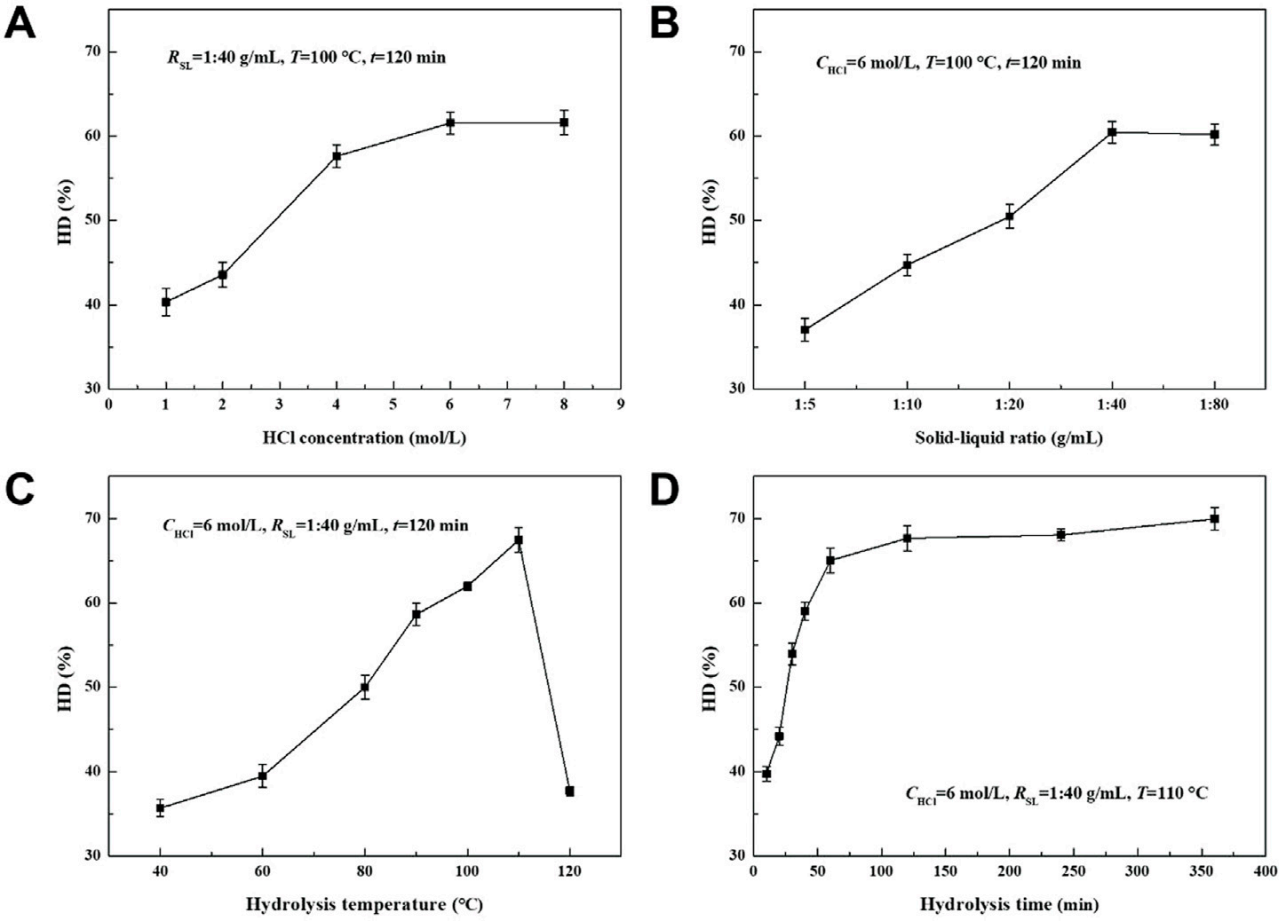
Effects of **(A)** HCl concentration, **(B)** solid-liquid ratio, **(C)** hydrolysis temperature, and **(D)** time on the HD of proteins.

Figure 2 shows the photographs of AFR before and after HCl hydrolysis. The hydrolysis performance of proteins in AFR was evaluated under different conditions, including *C*_HCl_, *R*_SL_, *T*, and *t*, as shown in Figure 3. Figure 3A shows the effect of *C*_HCl_ on the HD of proteins. The HD increased first and then tended to equilibrium with the increase of *C*_HCl_, ranging from 40.3% to 61.6%. This is because the amount of hydrogen ions increased with the increase of *C*_HCl_ until 6 mol/L, and the breaking of amide bonds in proteins was intensified. With the continuous increase of *C*_HCl_ after exceeding 6 mol/L, there are enough hydrogen ions, and the breaking was no longer intensified.

Figure 3B displays the effect of *R*_SL_ on the HD of proteins. The HD increased first and then tended to equilibrium with the increase of *R*_SL_, ranging from 37.0% to 60.4%. When the *R*_SL_ was low, the contact between hydrogen ions and amide bonds in proteins was inadequate, resulted in a low reaction efficiency. The contact was improved with the increase of *R*_SL_ until 1:40 g/mL. The contact was adequate and no longer improved when the *R*_SL_ exceeded 1:40 g/mL.

The effect of *T* on the HD of proteins is shown in Figure 3C. The HD first increased and then decreased with the increase of *T*, ranging from 35.7% to 67.5%. The maximum value was obtained at the *T* of 110°C. When the *T* was low, the reaction was slow with less energy in the reactants. The reaction was accelerated by increasing the *T*. However, HCl was evaporated and left the liquid when the *T* was higher than 110°C, resulted in a significant decrease in HD.

The effect of *t* on the HD of proteins is shown in Figure 3D. The hydrolysis reaction continued and the HD increased with *t* until equilibrium at about 120 min, and ranged from 39.7% to 70.0%. It can be seen that the reaction rate gradually decreased with *t* because of the consumption of reactants. According to the above results, the optimal conditions were obtained as *C*_HCl_ of 6 mol/L, *R*_SL_ of 1:40 g/mL, *T* of 110 °C, and *t* of 120 min. The HD of proteins of 67.7% can be achieved under these conditions, which was much higher than the HD of proteins in the literature (Ma et al., 2010; Hu et al., 2015; Dai et al., 2019).

#### 3.1.2 Correlation of hydrolysis degree

An empirical correlation of the HD of proteins in AFR, considering *C*_HCl_, *R*_SL_, *T*, and *t* was established to evaluate their contribution. The correlation was fitted based on the results of hydrolysis experiments in Section 3.1.1, as shown below:

$$\text{HD}=0.1405\times {\left(C_{\text{HCl}}+1\right)}^{0.40}\times R_{\text{SL}}^{0.25}\times T^{0.74}\times t^{0.20}\;\left(R^{2}=0.9703\right)$$ (1)

**FIGURE 4:**
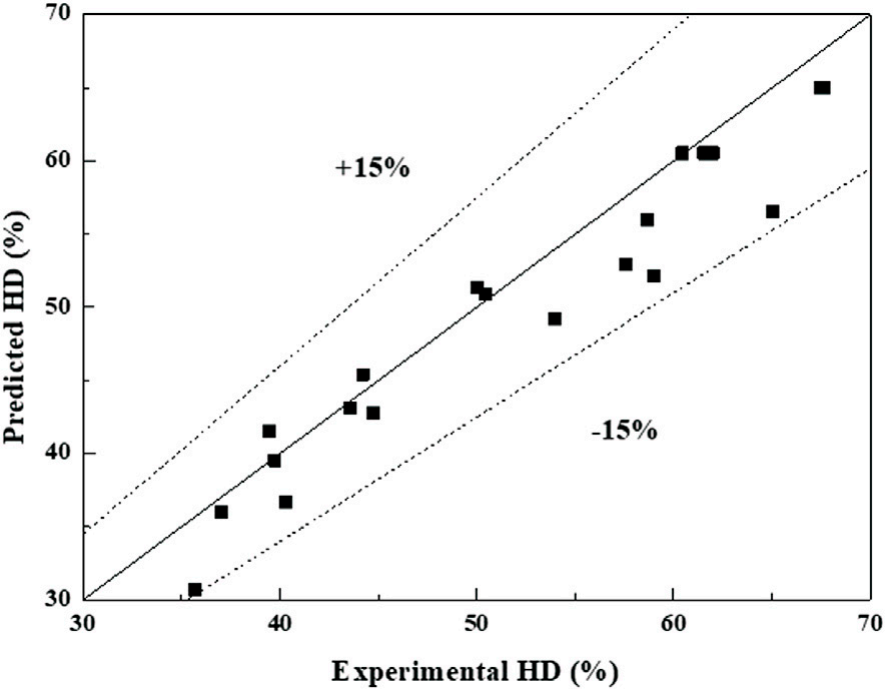
Comparison of HD between predicted and experimental values.

The predicted values agreed well with the experimental values with the errors of ±15%, as shown in Figure 4. This suggested that the correlation was practical to predict the HD in the range of 1–6 mol/L for *C*_HCl_, 1:5 to 1:40 g/mL for *R*_SL_, 40°C–110°C for *T*, and 10–120 min for *t*. The HD was proportional to the *C*_HCl_ +1, *R*_SL_, *T*, and *t* with the power of 0.49, 0.25, 0.66, and 0.19, respectively. This indicated that *C*_HCl_, *R*_SL_, *T*, and *t* all affected the HD. The higher the *C*_HCl_, the larger the *R*_SL_, the higher the *T*, the longer the *t*, and the higher the HD. The reasons were consistent with the analyses of HD in Section 3.1.1. The order of influence on the HD was *T* > *C*_HCl_ > *R*_SL_ > *t*. By the correlation, the required HD can be controlled and the experimental conditions can be calculated.

Experiment 1 and 2 were chosen to verify the correlation, and the experimental conditions and results are listed in Table 2. The errors between the predicted values and experimental values were 0.2% and 3.1%, respectively. This indicated that the correlation can be used to predict the HD with a high accuracy.

**TABLE 2:** Experimental conditions and results for the verification of correlation.

Experiment	Condition				Result	
	*C*_HCl_ (mol/L)	*R*_SL_ (g/mL)	*T* (°C)	*t* (min)	Predicted HD (%)	Experimental HD (%)
1	5	1:40	110	120	61.1	61.2
2	6	1:30	110	120	60.5	58.6

### 3.2 Analysis of amino acids in the hydrolysate

**FIGURE 5:**
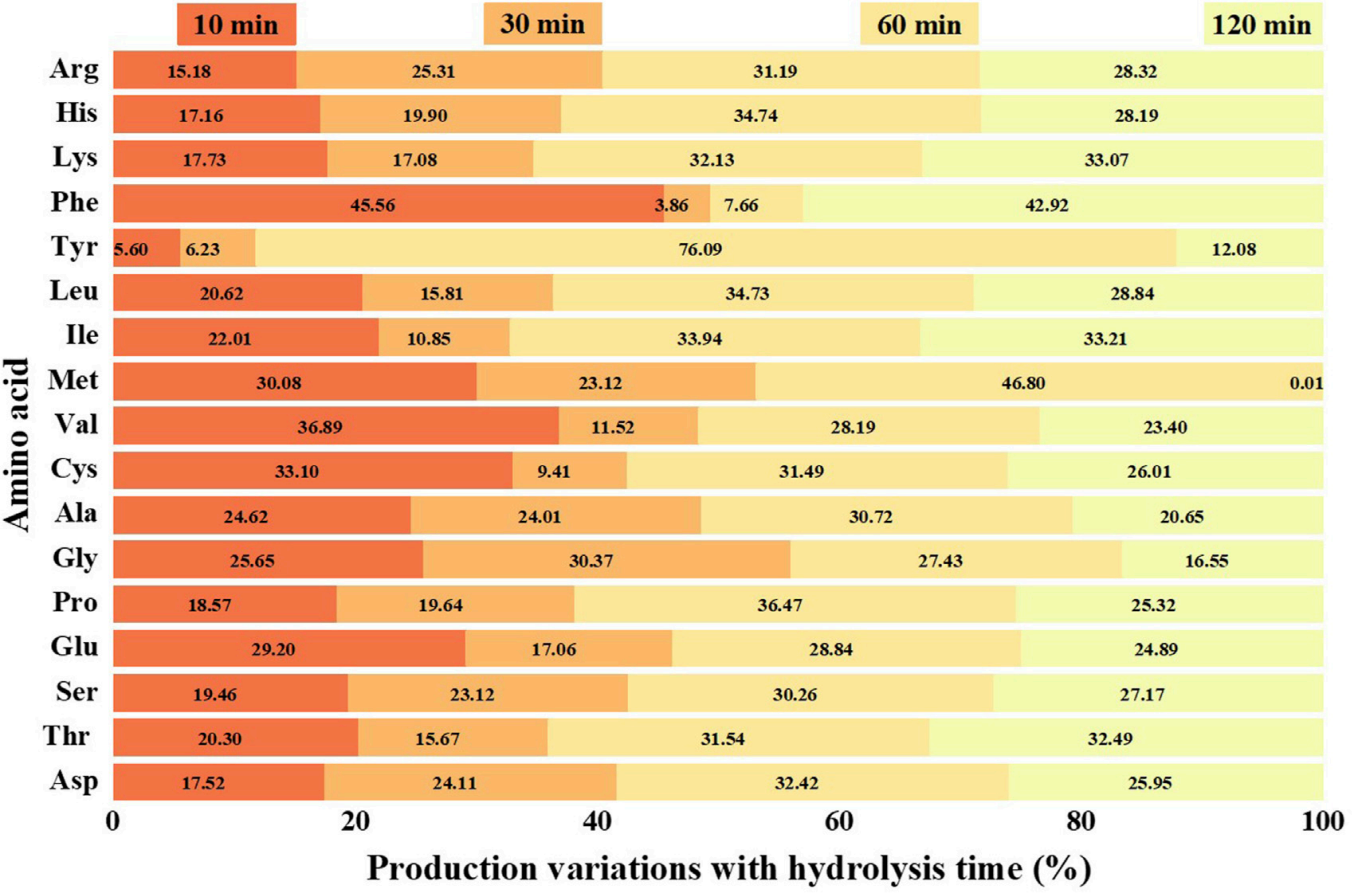
Amino acid production variations with hydrolysis time.

Through HCl hydrolysis of AFR, the proteins were converted into soluble amino acids. The amino acid kinds and contents in the hydrolysate under the optimal conditions were analyzed, as listed in Table 3. There were 17 kinds of amino acids in the hydrolysate, including glutamic acid (Glu), aspartic acid (Asp), alanine (Ala), phenylalanine (Phe), glycine (Gly), leucine (Leu), threonine (Thr), proline (Pro), serine (Ser), arginine (Arg), valine (Val), lysine (Lys), tyrosine (Tyr), isoleucine (Ile), histidine (His), methionine (Met), and cysteine (Cys). The amino acid production variations with *t* are displayed in Figure 5. The amino acid contents all increased with *t* until 120 min. The order of amino acid production rate in 10 min was Phe > Val > Cys > Met > Glu > Gly > Ala > Ile > Leu > Thr > Ser > Pro > Lys > Asp > His > Arg > Tyr. The order of production rate in 30 min was Gly > Met > Phe > Ala > Val > Glu > Ser > Cys > Asp > Arg > Pro > His > Leu > Thr > Lys > Ile > Tyr. The order of production rate in 60 min was Met > Tyr > Gly > Ala > Val > Glu > Pro > Asp > Cys > Ser > His > Arg > Leu > Thr > Lys > Ile > Phe. The differences in the production rates at different stages were mainly related to the structures of proteins in AFR. The concentration of different amino acids in the hydrolysate can be controlled by changing *t*, which was convenient for the selection of different amino acids. The production of value-added amino acids was beneficial to the in-plant reuse of AFR as nutrient sources. Among the amino acids, Thr, Ile, and Val can provide a significant improvement of the fermentation process for producing avermectin (De Rossi et al., 1995; Zhang et al., 1999; Cao et al., 2017).

**TABLE 3:** Amino acid kinds and contents in the hydrolysate.

Amino acid	Content (mg/L)	Amino acid	Content (mg/L)
Glu	144.0	Arg	49.1
Asp	104.7	Val	42.1
Ala	86.7	Lys	38.1
Phe	80.3	Tyr	30.8
Gly	79.7	Ile	22.1
Leu	74.7	His	19.5
Thr	58.1	Met	13.5
Pro	57.6	Cys	6.2
Ser	55.1	Total	962.3

### 3.3 Determination of critical reaction process

As described in Section 2.2, the hydrolysis performance of proteins affected by hydrolysis mode was investigated in this work. The HD of proteins for mode #2 and #3 at 120 min was 58.2% and 65.1%, respectively. According to the results in Figure 3D, the HD at 30 and 60 min was 54.0% and 65.0%, respectively, and the HD for mode #1 was 67.7%. For mode #2, the HD increased by 4.2% from 30 to 120 min, and for mode #3, the HD increased by 0.1% from 60 to 120 min. This indicated that a small number of proteins were dissolved in the liquid with HCl. The hydrolysis of proteins existed in both the solid residue and liquid at 30 min, and mainly existed in the solid residue at 60 min. The HD at 120 min for mode #2 and #3 was both lower than that for mode #1. Therefore, the solid-liquid reaction was dominant during the hydrolysis process. This is mainly because most proteins have the insoluble property, which was consistent with the description in Introduction. The hydrolysis process can be intensified by improving the solid-liquid reaction.

### 3.4 Analysis of other components in the hydrolysate

After HCl hydrolysis of AFR with the optimal conditions, the soluble saccharide content in the hydrolysate was measured as 1,628.0 mg/L, which was calculated as 65.1 mg/g in AFR. As shown in Table 1, the soluble saccharide content in AFR was 45.1 mg/g. This suggested that some insoluble saccharides were before hydrolysis depolymerized into soluble small molecules, which was beneficial to the in-plant reuse. The fats were not detected in the hydrolysate (<5 mg/g), indicating that the fats were depolymerized into glycerol and fatty acids under acid medium. The reduced mass of AFR after HCl hydrolysis was measured and reached 53.8 wt%. The significant decrease of mass means that the difficulty and cost of AFR treatment are largely reduced with the release of a large number of value-added small molecules.

### 3.5 Pathway of avermectin depolymerization

**FIGURE 6:**
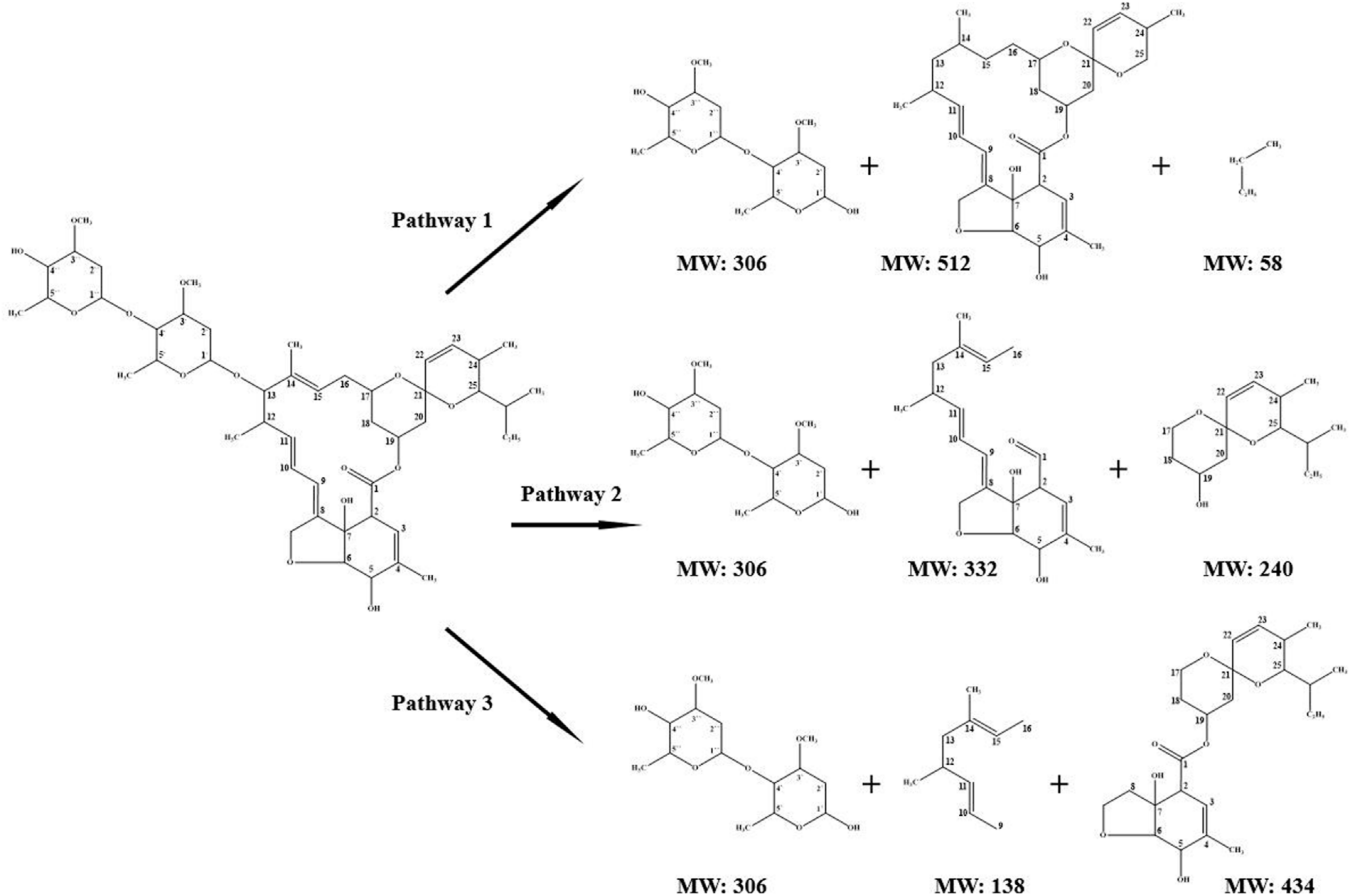
Schematic diagram of possible pathways of avermectin depolymerization.

As shown in Table 1, the avermectin content in AFR was 0.4 mg/g. After HCl hydrolysis of AFR with the optimal conditions, avermectin was not detected in the hydrolysate and AFR (<0.0006 mg/g), indicating an adequate depolymerization of avermectin. Table 4 presents the data of the products of avermectin depolymerization identified by LC-MS. The chemical formula and corresponding molecular weight (MW) were determined. The pathways of avermectin depolymerization were speculated, as shown in Figure 6. In pathway 1, C13-O and C25-C in avermectin (C_48_H_72_O_14_) were broken to form C_14_H_26_O_7_, C_30_H_40_O_7_, and C_4_H_10_. In pathway 2, C13-O, C16-C17, and C1-O were broken to form C_14_H_26_O_7_, C_20_H_28_O_4_, and C_14_H_24_O_3_. C13-O, C16-C17, and C8-C9 were broken to form C_14_H_26_O_7_, C_10_H_18_, and C_24_H_34_O_7_ in pathway 3. The depolymerization of avermectin may reduce the pollution risks during AFR treatment (Lv et al., 2021; Zhou et al., 2023). Acid hydrolysis provides the potential for environmentally friendly treatment and in-plant reuse of AFR.

**TABLE 4:** Data of the products of avermectin depolymerization identified by LC-MS.

Chemical formula	Molecular weight	Retention time (min)
C_10_H_18_	138	1.5
C_4_H_10_	58	3.4
C_30_H_40_O_7_	512	5.5
C_14_H_26_O_7_	306	7.5
C_20_H_28_O_4_	332	9.4
C_14_H_24_O_3_	240	9.8
C_24_H_34_O_7_	434	11.0

## 4 Conclusion

In this work, a novel strategy for HCl hydrolysis of AFR was proposed to release value-added small molecules. The conditions were optimized as HCl concentration of 6 mol/L, solid-liquid ratio of 1:40 g/mL, temperature of 110 °C, and time of 120 min, and the HD of proteins can reach 67.7%. The empirical correlation of HD was established, and the predicted HD agreed well with the experimental HD with the errors of ±15%. Proteins were hydrolyzed into 17 kinds of amino acids. The hydrolysis process existed in both the solid residue and liquid, and was dominant in the solid residue. In addition, there were 1,628.0 mg/L of soluble saccharides and undetected fats in the hydrolysate. The reduced mass of AFR after hydrolysis was 53.8 wt% with undetectable avermectin. The HCl hydrolysis was feasible with the advantages of high mass reduction and in-plant reuse of AFR.

## Figures and Tables

**Table:** 

***C***_**HCl**_	hydrochloric acid concentration (mol/L)
***R***_**SL**_	solid-liquid ratio of residues and hydrochloric acid solution (g/mL)
***T***	hydrolysis temperature (°C)
***t***	hydrolysis time (min)

## Data Availability

The original contributions presented in the study are included in the article/Supplementary material, further inquiries can be directed to the corresponding author.
